# Using R in Regulatory Toxicology

**DOI:** 10.17179/excli2022-5097

**Published:** 2022-08-22

**Authors:** Felix M. Kluxen, Signe M. Jensen

**Affiliations:** 1 ADAMA Deutschland GmbH, Cologne, Germany; 2 Department of Plant and Environmental Sciences, University of Copenhagen, Copenhagen, Denmark

**Keywords:** regulatory toxicology, statistics, continued education, software tutorial, R

## Abstract

Statistical analyses are an essential part of regulatory toxicological evaluations. While projects would be ideally monitored by both toxicologists and statisticians, this is often not possible in practice. Hence, toxicologists should be trained in some common statistical approaches but also need a tool for statistical evaluations. Due to transparency needed in regulatory processes and standard tests that can be evaluated with template approaches, the freely available open-source statistical software **R** may be suitable. **R** is a well-established software in the statistical community. The principal input method is via software code, which is both benefit and weakness of the tool. It is increasingly used by regulating authorities globally and can be easily extended by software packages, e.g., for new statistical functions and features. This manuscript outlines how **R** can be used in regulatory toxicology, allowing toxicologists to perform all regulatory required data evaluations in a single software solution. Practical applications are shown in case studies on simulated and experimental data. The examples cover a) Dunnett testing of treatment groups against a common control and in relation to a biological relevance threshold, assessing the test's assumptions and plotting the results; b) dose-response analysis and benchmark dose derivation for chronic kidney inflammation as a function of Pyridine; and c) graphical/exploratory data analysis of previously published developmental neurotoxicity data for Chlorpyrifos.

## Introduction

Toxicological hazard assessments consider dose-response evaluation of an adverse effect compared to concurrent control. Often the response is also compared to historical control data, for quality assurance, to determine biological relevance, and to address the statistical multiple comparison concern (Kluxen et al., 2021[24]). A crucial part of hazard characterization is statistical analysis, to identify potentially non-random effects, preferably in a holistic assessment combining statistical and biological plausibility (Kluxen and Hothorn, 2020[22]; Kluxen and Jensen, 2021[23]).

The hazard characterization point estimate or point of departure (POD) used in risk assessment (directly or modified by uncertainty factors), is a dose or concentration, that does not show a notable and relevant effect. Examples of PODs include the No Observed Adverse Effect Level (NOAEL) and the benchmark dose (BMD) with a pre-defined adverse response (BMR) or preferably the associated benchmark dose lower limit (BMDL). Whether an adverse effect is observed depends on experimental sensitivity or statistical power (Brescia, 2020[5]) because the identification of the effect dose above the NOAEL is often based on statistical testing. Conversely, a BMD is derived by dose-response modeling.

When the statistical analysis in study reports or registration documents lacks transparency or detail its value might be unnecessarily compromised. Accordingly, the European Food Safety Agency (EFSA) published a guidance to increase the quality of statistical reporting (EFSA, 2014[10]) and thus the value of submitted data, reports, analyses and interpretations.

The EFSA guidance outlines a reporting regime that may be incorporated into technical reports. But more importantly, it also states how a statistical analysis can be transparently described. Notably, the guidance requests the use of *interval estimates* and *graphical summaries* on descriptive statistics and *model diagnostics* of the performed statistical analysis. It “(encourages) a fully open and *transparent* approach”, a *statistical interpretation* of results, and *reproducibility* of all statistical procedures. 

The use of confidence intervals describes the estimated effect sizes more transparently than binary statistical tests (Wasserstein et al., 2019[33]) and their use is readily applicable in toxicology (Hothorn and Pirow, 2020[16]). However, some discussion and training on their use may be needed in the toxicological community; generally, a more evolved understanding of statistics may be needed for the involved scientists and regulators and a closer cooperation with statisticians may be helpful (Kluxen, 2020[20]).

How can a transparent exchange of statistical methods between the different stakeholders be facilitated and documented? Further, how can toxicologists become better trained in statistics and use suitable tools for assessment? The free open source statistics software **R** (R Core Team, 2020[29]) may be a suitable tool to address both issues. 

This manuscript shortly describes **R** and potential benefits for toxicologists by using **R**, for example, being able to conduct all necessary analysis in toxicology within one single software environment. First, we give a short introduction to **R** with a few notes on data handling. This is followed by an overview of the most common statistical approaches used in toxicology and their availability in **R**. The overview is supported by code and practical examples for easy implementation.

## Very Short R-Tutorial

There are many good introductions available for **R**, e.g. Crawley (2013[6]) or Field et al. (2012[12]). There are also books specifically focused on toxicology, e.g., Hothorn (2016[15]) and Ritz et al. (2019[30]), which, however, require some familiarity with the software. Other sources explain the application of Bayesian statistics (Kruschke, 2010[25]; McElreath, 2015[26]). Today, also video steaming services, such as *youtube *(https://www.youtube.com), offer a wide range of webinars and tutorials for **R** in general or for specific software extensions/packages.

The following section aims to provide some context for using the software and introduces **R** coding to enable the reader to run the case examples. 

### Data format

In laboratory practice, results are often stored in MS Excel (Microsoft, Redmond, WA, USA)-type software. Excel is both used as a database and a preliminary assessment tool to generate summary statistics, e.g., mean and standard deviations, and may also be used for plotting data. For statistical analysis, dedicated software is typically used; commonly point-and-click programs, such as SPSS (IBM) or Sigma Plot (alphasoft), where pre-defined statistical tests or assessments can be selected. Regulatory studies are often assessed according to statistical decision trees, i.e., where a main test, which is often an analysis of variance (ANOVA)-type test, is selected based on the outcome of pre-tests or assumption tests (Kluxen and Hothorn, 2020[22]). Naturally, there is also software that is very easy to use and conducts the pre-tests automatically which is very convenient in laboratory practice, e.g., ToxRat (ToxRat Solutions GmbH, Alsdorf, Germany). 

**Figure 1:**
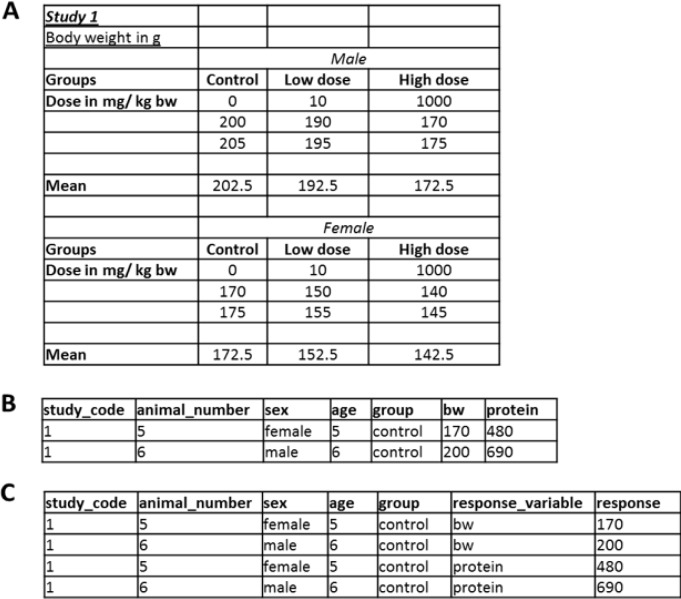
Data formats: While the format in (A) allows the comparison of groups, it is not useful for storing data. Preferred formats should be (B) or even more tidy (C), that are directly readable for most statistical programs and allow filtering in spreadsheet software.

For most statistical assessments and programs, data stored in Excel-type tabulating software has to be brought into a different format, typically from the so-called “wide” or unstacked format into the “long” or stacked format (Figure 1(Fig. 1)). For the latter, each measured response value is described by all variables resulting in that value, e.g., dose group, sex, time-point, etc. This format is not particularly handy to summarize or display data; hence, it is seldom available from laboratories (academic or commercial). However, the long format allows an easy and automatic stratification and handling of data and it is the typical structure of databases and the preferred data format for many statistical software programs. 

**R** can be used for data wrangling, statistical analyses, data exploration and presentation. While data formats can be changed with functions within **R**, data wrangling is very often a major part of any statistical analysis, thus, an effort to store data in a format optimal for statistical software is helpful.

### The statistical software R

**R** (R Core Team, 2020[29]) is a freely available open-source software based on the programming languages ***S*** and Scheme (Ihaka and Gentleman, 1996[17]). As such, the principal input is via a command line or a list of commands in a script, which is both the benefit and the weakness for the application in regulatory toxicology. 

**R** is very popular in the statistical community. Hence, **R** and its software extensions (named packages or libraries), which increase **R**'s functionality, are updated frequently. All types of statistical calculations can be performed or developed. Thus, there is no need to switch to dedicated software for some analysis, e.g., SPSS for Dunnett-testing, Excel for graphing and percentiles, and PROAST or BMDS for BMD derivation. All actions can be performed within one software, **R**. 

Due to its popularity and its coding background, solutions for a wide variety of programming issues are available. **R** comes with its own search engine (https://rseek.org), blogosphere (https://www.r-bloggers.com) and **R** questions are frequently featured in *stackoverflow *(https://www.stackoverflow.com/questions/tagged/r), a community question-and-answer website that rapidly (often within minutes) provides copy-paste solutions and has a searchable archive of previous problems.

**R** code can be either directly typed into a command line of the **R** console (the interface that is installed on the computer) or can be prepared as template code that may be stored in a file allowing re-using and sharing code from previous analyses in all details. Loss of knowledge from previous data assessment or data wrangling strategies helpful for future assignments is thus mitigated.

Upon installation, **R** consists of a *base system*, whose functionality can be enhanced according to the needs of the individual user, i.e., by custom functions. Many additional useful functions already exist and are bundled into packages, which can be downloaded from internet repositories, e.g., the Comprehensive **R** Archive Network (CRAN, https://cran.r-project.org) or github (https://github.com), and/or be distributed among colleagues. Packages from CRAN can be easily found and installed from within **R**. This is one main benefit of using **R**: everyone can program and build packages that can be made publicly available and peer-reviewed. Often when a new statistical method is developed the authors release an **R**-package along with the scientific publication. This makes new methods more approachable for others than statisticians and programming specialists. The downside is that the packages might be of varying quality, documentation and command syntax. Further, output formats may differ between packages, e.g., output from functions may be returned as numbers, lists or data frames with different nomenclature. This contributes to a relatively steep learning curve and frustration (*personal experience*), as compared to point-and-click software solutions. However, things have changed in the last few years. There are some concerted efforts to make data wrangling, exploration and analysis more intuitive, readable and harmonized, e.g., with the *tidyverse *(https://www.tidyverse.org) package/approach (Wickham et al., 2019[35]). Also, powerful graphical user interfaces (GUI) have been developed such as RStudio (https://rstudio.com), which gives suggestions, and auto completion of function names, etc., which tremendously increases **R**'s usability for both programmers and non-programmers. 

A major benefit of **R** is that a markdown language has been developed and conveniently integrated into RStudio. Markdown language makes it possible to easily combine text, code, analysis results and figures in one document. This makes the generation of “living documents”, i.e., reports in which the data is statistically assessed in-line, very easy and is consistent with the “reproducible research” paradigm (Gandrud, 2015[13]). Hence, a statistics document or annex itself can be conveniently generated with the **R** software environment.

**R **can be understood as an advanced calculator. Accordingly, one way to get started with **R** is to treat is as such. Below, some examples are given of how to slowly get started in R. > denotes input to be read by **R**. [1] denotes output/ results.

**Example R code from the R console..**

# everything to the right of “#” is a comment, comments are not run by R

> 1+2

[1] 3

> a <- 1 # assigning “<-” a value to an object “a”

> b <- 2

> c <- a + b # calculating with the contents of different objects and assigning the results to a new object “c”

> c # calling the object usually shows its contents 

[1] 3

> c^b - 2

[1] 7

> d <- c(a, b, c) # concatenating objects a-c and assigning this to a new object “d”

> d * 2 

[1] 2 4 6

> e <- c("a", "b", "c") # assigning a string vector to an object “e”

> f <- data.frame("Numbers"=d, "Names"=e) # creating a data frame out of objects “d” and “e”, with specified names

> f$NumbersDouble <- f$Numbers * 2 # “$” allows to interact with specific columns or vectors of the data frame

> str(f) # investigates the type of object and shows its contents

'data.frame': 3 obs. of 3 variables:

 $ Numbers : num 1 2 3

 $ Names : Factor w/ 3 levels "a","b","c": 1 2 3

 $ NumbersDouble: num 2 4 6

### Working with objects

A major advantage of using **R** is that it is an object-oriented language. Hence, the outcome of a calculation or an analysis can be assigned into a new object and used later, e.g., for further calculations or graphing. The user can conveniently work with or shift between several data sets or subsets in the same session.

**R** includes some publicly available standard datasets, which are often used as case studies in the **R** community. The available datasets can be viewed by using the *data()* command. Here we use the dataset ToothGrowth to illustrate a few **R** commands.

An Excel-like display can be achieved by using View(ToothGrowth) (note the capitalization, **R** is case sensitive). It shows the variables odontoblasts length in guinea pigs “*len*”, the Vitamin C dose (0.5, 1, or 2 mg/day) “*dose*” delivered in two forms, orange juice (*OJ*) or pure (*VC*) “*supp*”.

The dataset can be plotted using base **R** functions, plot(ToothGrowth), or, for example, after loading the package *tidyverse. tidyverse* uses a different syntax called “piping”, ToothGrowth %>% plot(), but both versions result in the same outcome. A better display can be achieved by specifying what should be plotted, plot(len~dose, data = ToothGrowth, col = supp), *i.e*., *len* explained by *dose *with both variables to be found in the data set* TootGrowth *and points should be colored according to the type of *supp*.

The beauty in **R** is that the objects can be re-used by other functions. As an example, we can combine functions to investigate how the mean of *len* changes by treatment and supplement type. 

library(tidyverse)

ToothGrowth %>% 

 group_by(dose, supp) %>% 

 summarize(mean_len = mean(len)) %>% 

 plot(mean_len~dose, data=., col=supp)

There are several ways how this can be achieved. However, the piping approach may be intuitively understood, which is important for future readers of the code. Often code, similar to notes in personal knowledge management, are revisited by its author later, hence, instructions and ideas should be formulated in a way that makes them understandable in the future (Ahrens, 2017[1]). Some things are easier using the piping notations; others are more convenient in base **R**. However, solutions for almost all use cases are available.

### Good working practice

One highly recommended GUI for **R** is RStudio. Here, data assessments can be collected in projects. This ensures that all necessary files for an analysis are within the same working environment. An **R**-project can be created within the folder where data are located. If a project and the data are transferred to a new location, the dependencies remain intact.

For repeatability of an analysis, it is convenient to source R-scripts. For example, a script containing code for loading the libraries needed for a specific analysis can be saved as “packages.R”, and can then be sourced in other scripts using source('packages.R') (note the single quotation marks).

**Code example of packages.R**

library(readxl) #to read Excel xls/xlsx files

library(tidyverse) #loading e.g. dplyr and ggplot2 for graphing

library(broom) #tidying up function output

library(multcomp) #for group-wise comparisons, e.g. Dunnett tests

library(sandwich) #to address heterogenous variances in the multcomp package

library(drc) # for dose-response analyses

library(bmd) # to estimate BMDs

### Data entry

Data can be entered by hand; however, usually data are imported from existing files. The following shows both approaches. First, a dataset is generated by sampling from a normal distribution, saved as an Excel file and imported from that same file (for illustration). This data set is similar to data set 1 presented in Kluxen and Jensen (2021[23]). Afterwards, data from a 13-week drinking water study of Pyridine in male F344/N rats are directly typed into **R**. The latter dataset contains information on doses, incidence of chronic kidney inflammation, total number of rats observed, mean (necropsy) body weight, and standard error of body weight (NTP, 2000[28]). The code on data entry can be saved as “datasets.R” for easy access to data in the case examples below.

**Generating example data**

source('packages.R')

set.seed(333111) #this ensures repeatability of the generated data

n <- 10 # a value is assigned to an object “n”

#generating normal distributed random data

response <- c(rnorm(n, 1, 0.5), rnorm(n, 1, 0.5),

 rnorm(n, 2, 1), rnorm(n, 1, 0.5),

 rnorm(n, 1, 0.5), rnorm(n, 5, 0.5)) 

#generating group allocations and concentrations

group <- c(rep(1, n),rep(2, n), rep(3, n),

 rep(4, n),rep(5, n), rep(6, n))

concentration <- c(rep(0, n), rep(10, n), rep(30, n),

 rep(100, n), rep(300, n),rep(1000, n))

#collecting the generated vectors/objects in a data frame

df1 <- data.frame(group, response, concentration, groupF = as.factor(group))

df1$groupF <- factor(df1$group) #new data type column to handle “group” as a factor

write.csv(df1, "df1.csv") 

**Data entry from file**

# example how to read in some Excel file: df_excel <- read_excel("example.xlsx")

df1_readFromFile <- read.csv("df1.csv")

**Typing in data**

pyridine <- as.data.frame(cbind(Dose = c(0, 50, 100, 250, 500, 1000),

 Inflammation = c(0, 0, 0, 2, 4, 9),

 Total = c(10,10,10,10,10,10),

 BW = c(335, 334, 337, 334, 316, 287),

 BW.SE = c(9, 7, 6, 7, 5, 5)))

## Further Examples and Case Studies

In the following, some common examples from regulatory practice are given to show how **R** is used for statistical assessments. 

### Dunnett testing

The Dunnett test (Dunnett, 1955[8]) is the most common statistical test in toxicology, as it compares treatment groups with a concurrent control. It can be modified to address various data conditions and is formulated as a contrast test, which is among others implemented in the package *multcomp* (Hothorn, 2007[14]). Here, the function “general linear hypothesis test” *glht()* can be used on a simple linear model, *lm()*. The *glht()* function is powerful and also works with more complex models, e.g., generalized linear models (*glm()* function of **R** base), and mixed-effects models (*lmer()* of the package *lme4* (Bates et al., 2015[3])) and it includes various other contrast tests, such as the Tukey, Williams or even custom contrasts. 

**Dunnett test example**

source('packages.R')

df1 %>%

 lm(response~groupF, .) %>%

 glht(., linfct=mcp(groupF="Dunnett"), alternative="two.sided") %>%

 summary()

Simultaneous Tests for General Linear Hypotheses

Multiple Comparisons of Means: Dunnett Contrasts

Fit: lm(formula = response ~ groupF, data = .)

Linear Hypotheses:

 Estimate Std. Error t value Pr(>|t|) 

2 - 1 == 0 -0.2099 0.2483 -0.846 0.8662 

3 - 1 == 0 0.7446 0.2483 2.999 0.0177 * 

4 - 1 == 0 -0.3331 0.2483 -1.342 0.5353 

5 - 1 == 0 -0.1578 0.2483 -0.636 0.9530 

6 - 1 == 0 3.6059 0.2483 14.523 <1e-04 ***

---

Signif. codes: 0 '***' 0.001 '**' 0.01 '*' 0.05 '.' 0.1 ' ' 1

(Adjusted p values reported -- single-step method)

The outcome would suggest that the null hypothesis could be rejected for the comparisons 3-1 and 6-1, as the p-values are not compatible with the assumption of null difference. 

In order to use confidence/compatibility intervals to estimate effect sizes, a re-assessment of an existing Dunnett analysis may be necessary. In **R**, the *summary()* function above, can simply be replaced by *confint()* with a specified confidence level, e.g., 90 % for two-sided comparisons at 5 % alpha each. This allows to assess how a specified value, representing biological relevance, relates to the effect size. 

**Dunnett test confidence intervals**

df1%>%

 lm(response~groupF, .) %>%

 glht(., linfct=mcp(groupF="Dunnett"), alternative="two.sided") %>%

 confint(level = 0.9)

Simultaneous Confidence Intervals

Multiple Comparisons of Means: Dunnett Contrasts

Fit: lm(formula = response ~ groupF, data = .)

Quantile = 2.289

90% family-wise confidence level

Linear Hypotheses:

 Estimate lwr upr 

2 - 1 == 0 -0.2099 -0.7783 0.3584

3 - 1 == 0 0.7446 0.1763 1.3129

4 - 1 == 0 -0.3331 -0.9014 0.2352

5 - 1 == 0 -0.1578 -0.7261 0.4105

6 - 1 == 0 3.6059 3.0375 4.1742

### Assessing model assumptions

Confidence intervals of the Dunnett contrasts assume that the model's residuals follow a normal distribution and that the variance associated with the effect size remains constant (variance homogeneity), i.e., it is not affected by treatment itself. These assumptions may or may not be appropriate or helpful. For some data types, e.g., count data, the assumptions may be *per se* incorrect, however, might still estimate the effect size in a way that is useful for a toxicological assessment. This might be the case for high numbers of counts with no zero observations. However, usually, the variance of count data is expected to change with the mean (this is the inherent assumption for logistic and Poisson distributions).

Model assumptions may be checked by statistical testing or (perhaps preferably) by visual assessments. 

**Statistical assessment of model assumptions**

# Shapiro-Wilk test for normality of residuals

df1 %>%

 lm(response~groupF, .) %>%

 resid() %>%

 shapiro.test()

# Bartlett test for homogeneity of variance

df1 %>%

 bartlett.test(response~groupF, .)

**Graphical assessment of model assumptions**

# Quantile-quantile plot for assessing normality of residuals

df1 %>%

 lm(response~groupF, .) %>%

 plot(., which=2)

# Residual plot for assessing variance homogeneity

df1 %>%

 lm(response~groupF, .) %>%

 plot(., which=1)

**Figure 2:**
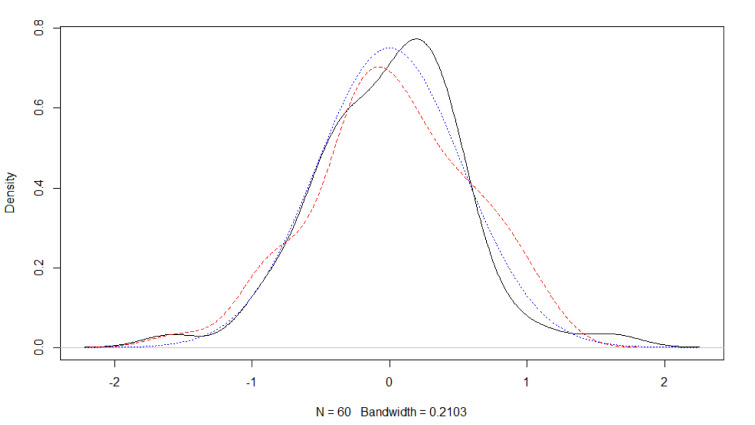
Assessing model assumptions with graphical methods of base R. Density of the residuals of a fitted model based on normal distributed values (black solid line). Density of values from a normal distribution with number and standard deviation identical to the residuals of the fitted model (red dashed line). Normal distribution with standard deviation identical to the residuals of the fitted model (blue dotted line).

Obviously, it is also possible to explore other approaches to investigate distributional assumptions with **R**. For example, by means of a miniature simulation study and graphical assessment (Figure 2(Fig. 2)). Here, the simulated data deviates in a similar fashion from the (perfect) normal distribution curve as the empirical residual distribution of dataset 1. Hence, one may consider the normal distribution assumption of the linear model's residuals supported.

**Checking for normal distribution of residuals from a linear model**

set.seed(654654) #allows exact replication of the example plot

fit <- df1 %>%

 lm(response~groupF, .) # linear model

normalData_sampled <- rnorm(length(fit$residuals), mean = 0, sd = sd(fit$residuals))

x <- seq(from=-3, to=3, length.out = 100)

plot(density(fit$residuals), main = "")

lines(density(normalData_sampled), col="red", lty=2)

curve(dnorm(x, mean = 0, sd = sd(fit$residuals), log = F), col="blue", lty=3, add = T)

# the default R plotting output can be saved by a “graphics” device which is wrapped around the plotting function

png("density.png", width = 10, height = 10, units = "cm", res=300)

→ INSERT plotting function(s)…

dev.off()

### Plotting Dunnett confidence intervals

*multcomp* objects can be plotted to visualize confidence intervals, by simply piping the *plot()* function after the *confint()* function above. However, the plot shows the contrasts, e.g., effect size change as compared to control mean. There are multiple ways to change this to the original scale - which can be helpful to assess model assumptions, e.g., homogeneous variance (Kluxen and Jensen, 2021[23]).

**Figure 3:**
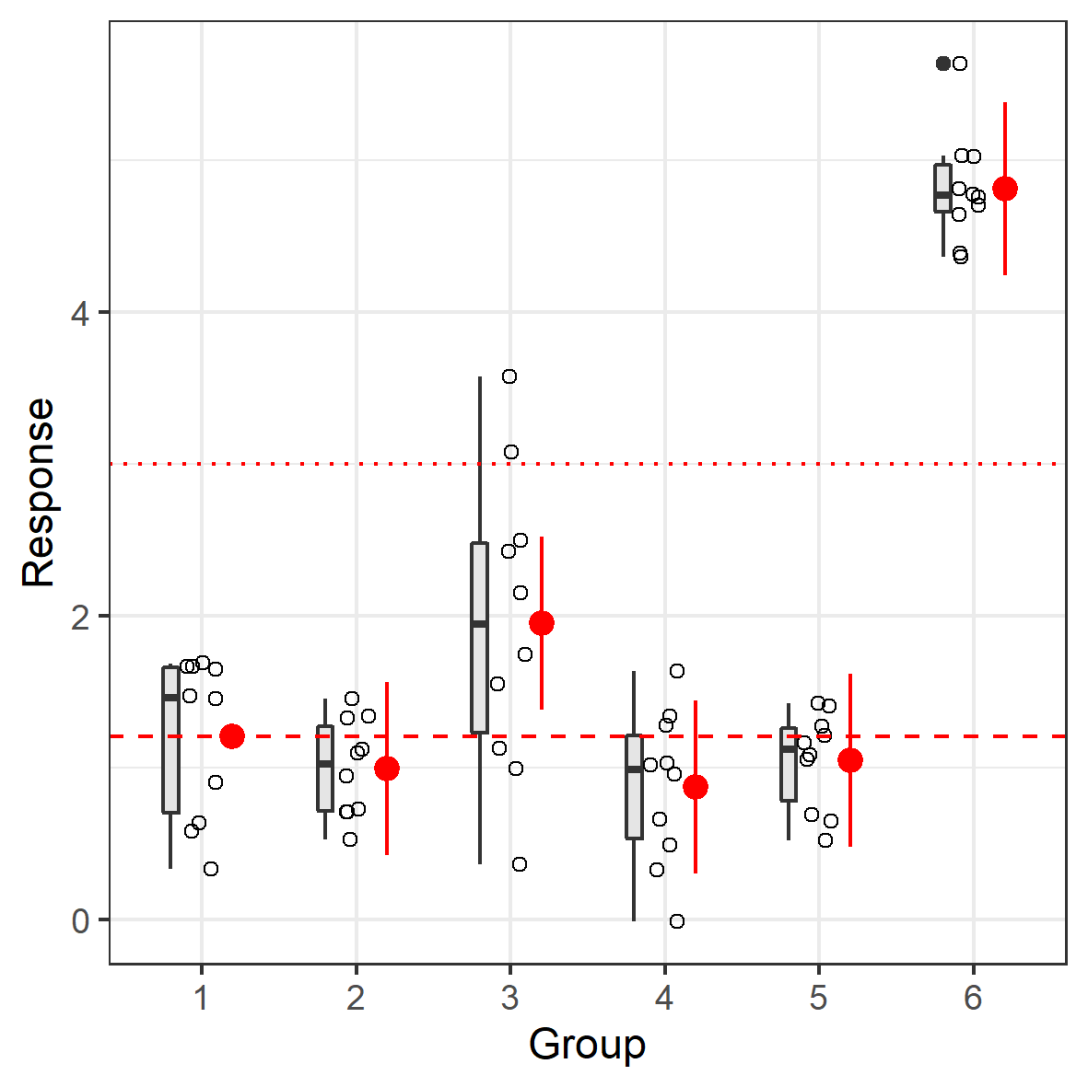
Simulated data plotted along with boxplots and Dunnett effect sizes. The dashed horizontal line shows the mean of group 1. The dotted red line could be a relevant effect size.

The following shows one way to plot confidence intervals along with the original data with the very customizable *ggplot2* package (Wickham, 2016[34]); overplotted is a horizontal line that could illustrate a biologically relevant response size (Figure 3(Fig. 3)). The confidence interval can enhance a toxicological interpretation (Hothorn and Pirow, 2020[16]; Kluxen, 2020[20]).

The ggplot objects that are created by the package consist of multiple layers, i.e., data and so-called geoms. In principle, a geom could be developed for the confidence interval function of glht(), which would make the following redundant. However, it shows how freely one can work with **R** objects between packages. The plot is rather busy, but it shows the possible display options by added geoms.

This visualization shows that the confidence intervals have the same size, independent of the actual variation in the groups. The Dunnett tests pools the variation of all groups; it assumes homogenous variance (which is the assumption/method of the underlying linear model). While the variation is increased in group 3, one could argue that there is no systematic change of variance, i.e., increase or decrease with treatment if groups 1-6 represent increasing treatment levels. Hence, one might consider the homogenous variance assumption to be appropriate. If not, one can adjust for heterogenous variance by using a sandwich estimator in *glht()* by adding the vcov=*sandwich* option (of the package *sandwich*), which would adapt the confidence interval sizes. 

**Dunnett test confidence interval visualization**

# creating a dataframe for the effect sizes

Dunnett <- df1 %>%

 lm(response~groupF, .) %>%

 glht(., linfct=mcp(groupF="Dunnett"), alternative="two.sided") %>%

 confint(level = 0.9) %>%

 tidy() %>% # creating a dataframe from the model output

 # adding the control mean to get intervals on original scale

 # groupF needs to be indexed in df1, as the confint dataframe does not contain it

 mutate(estimate=estimate+mean(response[df1$groupF==1]),

 conf.low=conf.low+mean(response[df1$groupF==1]),

 conf.high=conf.high+mean(response[df1$groupF==1])) %>%

 add_case(estimate=mean(response[df1$groupF==1]), .before = 1) %>% #adding control

 mutate(groupF=levels(df1$groupF))

#plot data and effect sizes 

df1 %>%

 ggplot(aes(groupF, response)) +

 geom_boxplot(fill="grey90", width=0.1, position=position_nudge(x=-0.2))+

 geom_point(position = position_jitter(width=0.1), shape=1)+ 

 geom_pointrange(data=Dunnett, aes(x=groupF, y=estimate, ymin=conf.low, ymax=conf.high), 

 color="red", position=position_nudge(x=0.2))+

 geom_hline(data=Dunnett, aes(yintercept=estimate[groupF==1]), color="red", 

 linetype="dashed")+

 geom_hline(yintercept=3, color="red", linetype="dotted")+

 labs(x="Group", y="Response")+

 theme_bw()

#ggplot2 output can be saved by a specific function. Either the plot is saved into a specific object and referenced or the last ggplot called from memory with the last_plot() function. 

ggsave("ggplot-output.png", width=10, height=10, units="cm", plot = last_plot())

The assumptions of the linear model can be explored graphically (Kluxen and Hothorn 2020[22]), for example by simply using the *plot()* function on the model, however, other approaches are possible (see above). 

### Dose-response analysis

**Figure 4:**
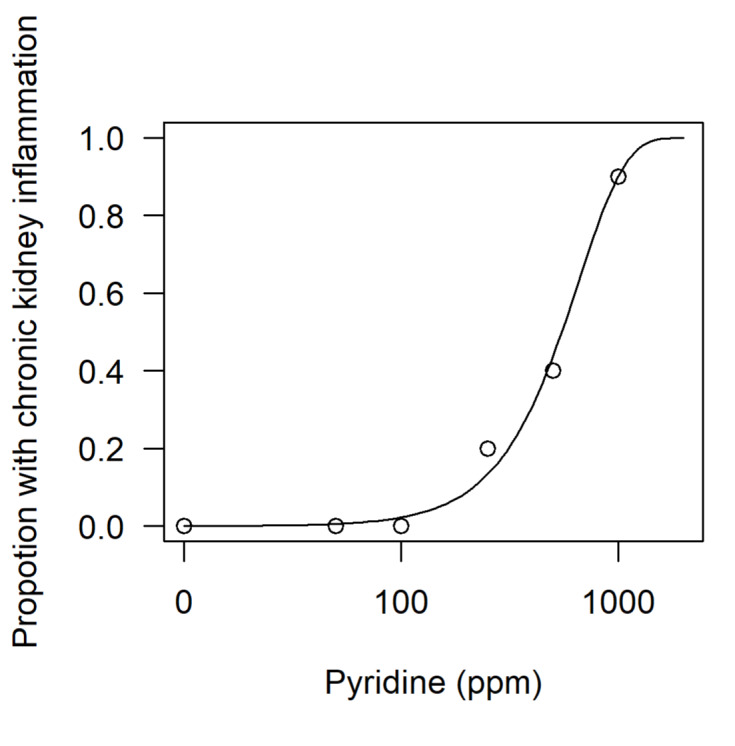
Default plot of the drc package

Fitting a dose-response model to data, e.g., chronic kidney inflammation as a function of Pyridine, can be done using the package *drc* (Ritz et al., 2019[30]). As for the *lm()* function, *drm()* requires a specification of the response to be explained by the dose, and further arguments telling **R** that these are binomial data and what type of dose-response model should be used. The build-in simple plot functionality shows the fitted model (Figure 4(Fig. 4)).

**Dose-response analysis binomial data example**

source('packages.R')

pyridine %>%

drm(Inflammation/Total ~ Dose, data =. , weights = Total, type = "binomial", fct = W2.2()) %>%

plot(., xlim = c(0,2000), ylim = c(0,1), 

ylab = "Propotion with chronic kidney inflammation", 

xlab = "Pyridine (ppm)") 

#the “.” Pipes the output of the previous function to the current function, it is needed for some functions that do not utilize piping by default

**Figure 5:**
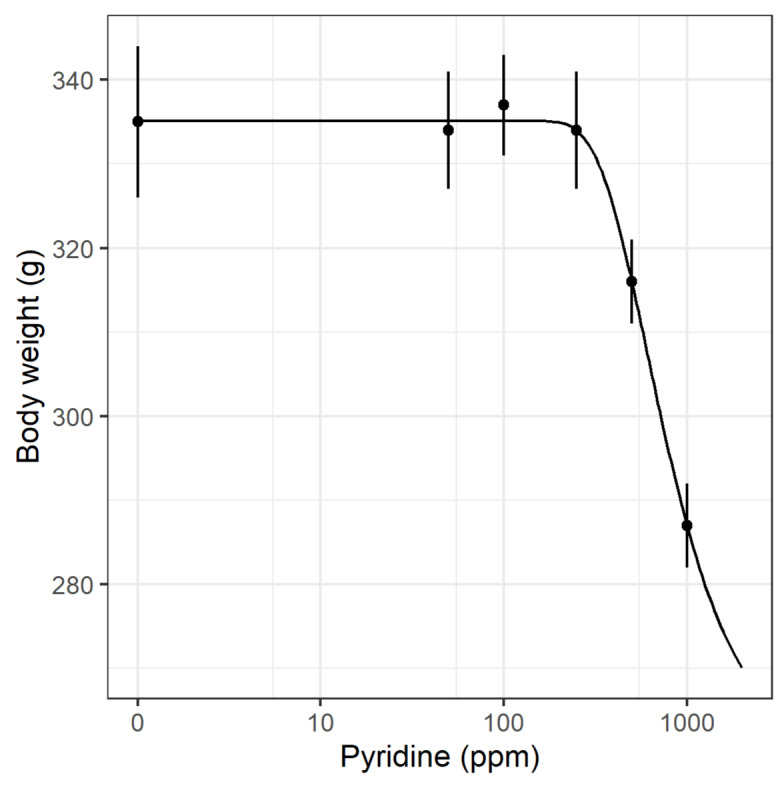
ggplot of a drc object

Using body weight as response variable, similar code provides a fitted dose-response model, and with a few more lines of code, a ggplot figure is obtained (Figure 5(Fig. 5)).

**Dose-response analysis continuous summary data example**

model.BW <- drm(BW ~ Dose, data= pyridine , weights = BW.SE, fct = W2.4()) 

newdata <- expand.grid(Dose = exp(seq(log(1), log(2000), length=100)))

pm <- predict(model.BW, newdata = newdata, interval="confidence")

newdata$p <- pm[,1]

newdata$pmin <- pm[,2]

newdata$pmax <- pm[,3]

pyridine$Dose0 <- pyridine$Dose

pyridine$Dose0[pyridine$Dose0==0] <- 1

ggplot(pyridine, aes(x=Dose0, y=BW))+

 geom_point()+

 geom_errorbar(aes(ymin=BW - BW.SE, ymax=BW + BW.SE), width=0)+

 geom_line(data=newdata, aes(x=Dose, y=p))+

 coord_trans(x="log") + 

 ylab("Body weight (g)")+

 xlab("Pyridine (ppm)")+

 theme_bw()+

 scale_x_continuous(breaks=c(1,10,100,1000),label=c(0,10,100,1000))

One disadvantage of *drc*, which may however also be seen as a strength, is that the user must decide on both the type of dose-response model to go with and the number of parameters. For the binomial data example, i.e., chronic kidney inflammation, a 2-parameter Weibull model was chosen as both lower and upper limits were assumed known to be 0 and 1, respectively. For the continuous data, no previous knowledge on any of the parameters were assumed and a 4-parameter Weibull model was therefore selected. When previous knowledge on the dose-response shape is known, this can be utilized in the model fitting by “fixing” one or more parameters, e.g., the 2-parameter Weibull model is a pre-coded version of the 4-parameter model where lower and upper limit is fixed. The result is a model where the remaining parameters are fitted with more precision, i.e., lower standard errors.

### Benchmark dose evaluation

Given a dose-response model fitted using *drc*, the package *bmd* can be used for estimating BMD and BMDL (Jensen et al., 2020[18]). For the binomial data example above, an obvious choice could be to estimate BMD using the “*excess risk*” definition and a pre-defined BMR=0.1, while the “*relative*” definition of BMD corresponding to estimating the critical effect size could be the choice for the continuous data (Slob, 2017[31]). The BMDL based on bootstrapping, using the *bmdBoot()* function, involves random sampling. For reproducibility, it is important to “set a seed”. 

**BMD analysis for binomial data**

set.seed(1001)

pyridine %>%

 drm(Inflammation/Total ~ Dose, data=. , weights = Total, 

 type="binomial", fct=W2.2()) %>%

 bmdBoot( . , 0.1, backgType="modelBased", def="excess") BMD BMDL

 215.4948 144.5428

**BMD analysis for continuous data**

set.seed(1001)

pyridine %>%

 drm(BW ~ Dose, data = . , weights = BW.SE, fct = W2.4()) %>%

 bmdBoot( . , 0.1, backgType="modelBased", def="relative")

 BMD BMDL

 691.3295 497.8342

### Real case example with Chlorpyrifos

A real case example might be helpful to demonstrate how **R** can be used to re-assess data for toxicological insight.

Mie et al. (2018[27]) previously reviewed a regulatory developmental neurotoxicity (DNT) study conducted with the insecticide Chlorpyrifos. It was suggested that changes in cerebellum height and cerebellum height relative to brain weight would indicate a DNT effect for Chlorpyrifos. While there appeared no effects in the high-dose group for cerebellum height relative to brain weight, the effects in the low- and mid-dose groups were pronounced and statistically significant. Juberg et al. (2019[19]) previously refuted the assessment of Mie et al. (2018[27]). Unfortunately, the data set cannot be shared with the readers, as it is propriatary information not owned by the authors' institutions. However, the approach itself is generic and suitable for similar cases.

**Figure 6:**
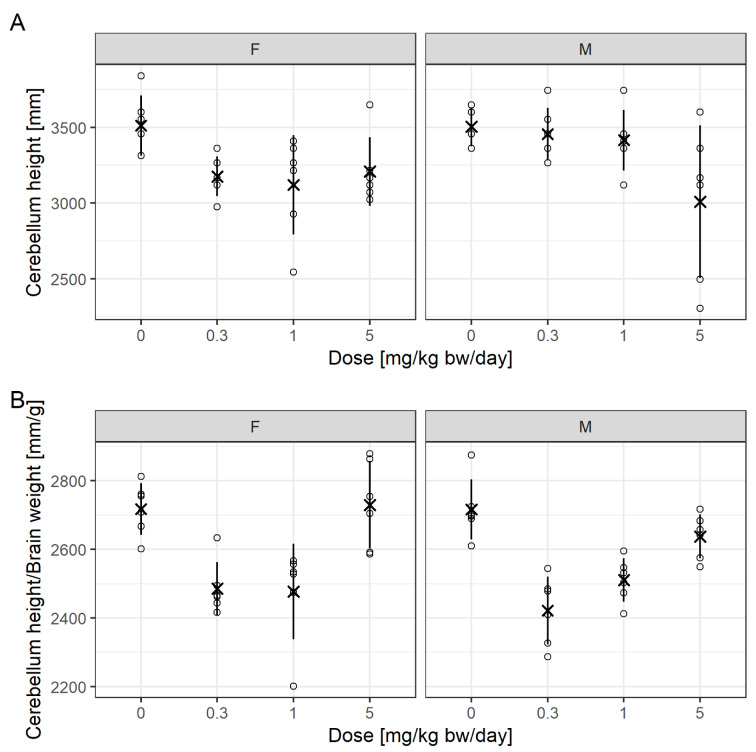
Data from Mie et al. (2018), showing cerebellum height and cerebellum height to brain weight over dose of Chlorpyrifos, with individual values (open circles) added along with mean values (multiplication sign) and standard deviation (line range), which can be added by applying a custom function.

Figure 6(Fig. 6) is reproduced from Mie et al. (2018[27]) and now also includes individual values. Notable are extreme values and differences in variation for cerebellum height between treatment groups for males, driven by two animals. Superimposed are mean values and the standard deviation by adding a custom layer to the normal scatter plot from ggplot2. This approach allows the addition of summary statistics relevant to the assessment, others could be percentiles.

**Adding a custom function to a ggplot2**

 stat_summary(geom = "pointrange",

 fun = "mean",

 fun.min = function(x) mean(x) - sd(x),

 fun.max = function(x) mean(x) + sd(x),

 color="black", shape=4)

For Figure 6A(Fig. 6), Mie et al. (2018[27]) reported that the 1 mg/kg bw/day dose was statistically significantly different to control for females and the 5 mg/kg bw/day different to control for males, according to a Dunnett test. 

For Figure 6B(Fig. 6), the 0.3 and 1 mg/kg bw/day doses were different to control for both sexes, while the top dose was not. 

The previous statistical analysis is not replicated here for the following reasons. 

1) For cerebellum height, the homoscedasticity assumption might be scrutinized, at least for males. 

2) Cerebellum height to brain weight is a ratio, which can be easily biased by effects on either factor and when they correlate the assessment of their ratio may become meaningless (Curran-Everett, 2013[7]). In fact, the curious u-shaped dose-response and the lack of effect for the high-dose group for both sexes strongly suggest the presence of a confounding effect. 

3) There is an issue with generic statistical testing as a single decision criterion (Wasserstein and Lazar, 2016[32]), especially when using small group sizes where individual response values have much influence on the assessment. 

4) In practice, it makes sense to compare responses not only to the concurrent control mean but also to relevance thresholds, for example based on historical control data, on the one hand to identify biological relevance of effects and on the other to address the multiple comparison problem. Unfortunately, historical control data for cerebellum height are scarce. 

5) There is the limitation that the litters of the F1 generation pups cannot be reproduced from the data available to the authors. There might be a genetic predisposition with regard to brain morphology, which is not captured or accounted for in a classical Dunnett-type statistical analysis. Hence, it is unclear whether the extreme responses are observed in pups that come from the same litter and whether the variation is appropriately modeled by a simple linear model. For example, the animal numbers of the minimum and maximum response for cerebellum height in the high-dose male group are 104 and 108, respectively. Considering subsequent numbering and a mean litter size of 13 pups/litter in that treatment group, the pups are likely to come from the same litter. A mixed-model that consideres both within and between litter variation would then presumably not find a significant effect for that treatment group for cerebellum height. Also, if the historical background variation is not considered, it is unclear whether such a variation is in itself a toxicologically relevant effect or common/normal.

Due to this uncertainty, a more abstract and holistic approach may be applied. This may have benefits if statistical model assumptions are violated (Kluxen and Jensen, 2021[23]). Ratios and confounding can be investigated by simple scatterplotting, as proposed for investigating relative organ weights (Kluxen, 2019[21]) but can also be applied on other measures (Bomann et al., 2021[4]). This approach has the obvious limitation that no associated uncertainty is estimated for the assessment.

**Scatterplotting to investigate rates, confounders or relative organ weights**

chlorpyrifos %>%

 ggplot(aes(x=body_weight, y= organ_weight, color=Dose, shape=Dose)) +

 geom_point()+

 facet_grid(~Sex)

**Figure 7:**
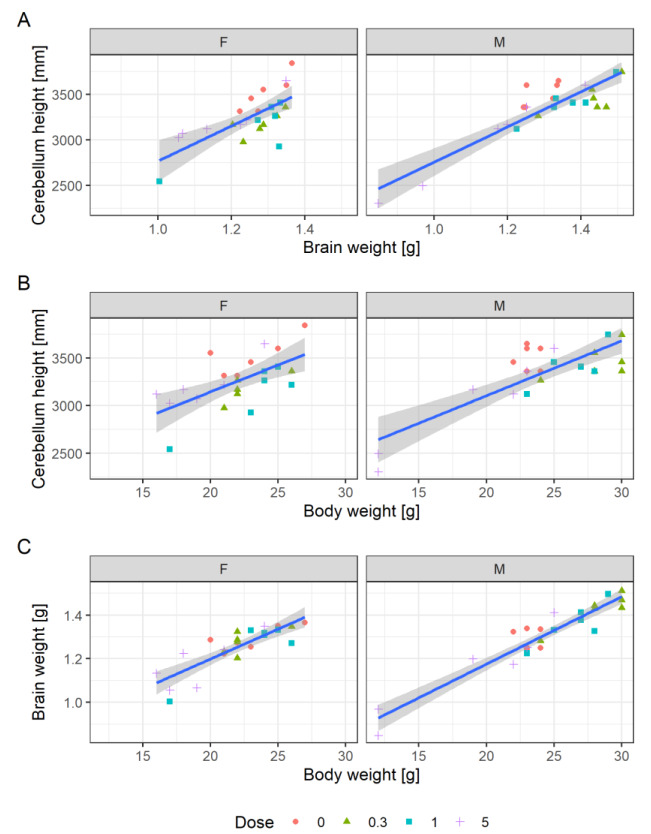
(A) Cerebellum height to brain weight, (B) cerebellum height to body weight, and (C) brain weight to body weight scatter plot, overplotted with linear models. The dose levels are depicted as symbols.

Figure 7A(Fig. 7) shows a scatterplot of cerebellum height to brain weight. The individual values indicate a direct correlation: animals with a high brain weight also have a higher cerebellum height. The plot shows that the two male high-dose animals that have the lowest cerebellum heights also have the lowest brain weights.

Figure 7B(Fig. 7) shows a scatterplot of cerebellum height to body weight and (C) brain weight to body weight. The animals with the lowest cerebellum height and brain weight also have the lowest body weight. A simple linear model for the correlation of body and brain weight, ignoring sex and dose, explains 84 % of the variance. The model can be superimposed in the plot by adding: geom_smooth(method="lm", aes(group=1)). This model does not seem to explain more of the available variation when additionally considering dose as a cofactor. 

**Simple comparison of linear models**

fit_1 <- chlorpyrifos %>% 

 lm(Brain_wt~Body_wt, .)

fit_2 <- chlorpyrifos %>% 

 lm(Brain_wt~Body_wt+as.numeric(Dose), .) 

anova(fit_1, fit_2)

Analysis of Variance Table

Model 1: Brain_wt ~ Body_wt

Model 2: Brain_wt ~ Body_wt + as.numeric(Dose)

 Res.Df RSS Df Sum of Sq F Pr(>F)

1 46 0.1286 

2 45 0.1264 1 0.0022034 0.7845 0.3805

Hence, since cerebellum height, brain weight and body weight correlate, an effect on body weight is propagated through the endpoints. This effect is exacerbated when endpoints are assessed in ratios and results in curious dose-response relationships, as seen for cerebellum height to brain weight ratio. 

Here, animals with notably lower cerebellum heights are clearly smaller than other animals. This observation casts doubt on a targeted effect on brain morphology but supports an effect following general maternal toxicity. The relationship of body weight and organ weight is complex (Bailey et al., 2004[2]; Kluxen, 2019[21]) and driven, for example, by the need for a certain organ size to ensure physiological function. This may be even more relevant for the brain and associated endpoints. 

## Discussion

We hope that we convinced the reader that **R** gives its user the technical means to conduct all common statistical assessments within the same tool. Especially BMD modeling is usually conducted in dedicated software. This flexibility is convenient and contributes to transparency, as all assessments can be communicated by the same canonical software code. The graphical extension ggplot2 allows high quality 2d plots of most cases relevant in toxicology.

There are additional software extensions available that may be suitable for toxicological analysis. For example, for toxicokinetic-toxicodynamic (TKTD), pharmacokinetic and pharmacodynamic (PB/PD) modeling or survival analysis. 

Regulatory authorities have a documented use of **R**, for example for deriving default values for dermal absorption (EFSA, 2017[9]), as exposure models (EFSA, 202[11]2), or as the basis for BMD modelling (https://github.com/NIEHS/ToxicR, https://www.rivm.nl/en/proast). Hence, one can imagine that there is in-house competence to use R-scripts. An **R** package was recently successfully submitted to the United States Food and Drug Administration (US FDA) as an electronic Common Technical Document (eCTD, r-consortium.org/blog/2021/12/08/successful-r-based-test-package-submitted-to-fda), demonstrating transparency and reproducibility of analyses. This also demonstrates that there is a certain need for toxicologists to built-up some basic competences in **R** in order to keep up with such developments.

When older studies are used in novel registration procedures, and new methods for statistical assessment are available or recommended, e.g., effect size estimation as compared to statistical hypothesis testing, documented **R** code can be easily adjusted for new requirements or methods by all stakeholders in registration processes. 

The code-type input in **R** may have a steep learning curve and inconsistencies between function grammars can be confusing. The user also encounters normal programming issues, for example, that a missing comma or parentheses disrupt a complete analysis. However, the tool allows a toxicologist to perform and replicate virtually all possible statistical assessments. It may further serve as a common language between statisticians and toxicologists. By this, **R** may aid in the statistical education of toxicologists. Having to write code for an analysis may also urge the toxicologist to explore different routes, e.g., graphical inspection of residuals of a fitted model to assess normal distribution instead of conducting generic testing (Kluxen and Hothorn, 2020[22]). 

## Conclusion

**R** is a powerful and free open-source tool that may be very valuable for toxicologists by allowing all regulatory required data evaluations in one software. Toxicologists are empowered to perform their own statistical evaluations and communicate those with statisticians and authorities. 

## Conflict of interest

The authors declare that they have no conflict of interest. FMK works as a scientific expert in regulatory toxicology for ADAMA, which distributes and markets pesticides.
